# Digital Attention Bias in Cancer Survivors Intervention for Adolescent and Young Adult Cancer Survivors: Protocol for a Pilot Randomized Controlled Trial

**DOI:** 10.2196/82665

**Published:** 2026-02-25

**Authors:** Nancy Lau, Chuan Zhou, Shannon JH Hong, Homer Aalfs, Shannon Higgins, Isabel Badillo, Elizabeth McCauley, Tyler Ketterl, Eric J Chow, Jesse R Fann, Lauren C Heathcote, Tonya M Palermo

**Affiliations:** 1 Department of Psychiatry and Behavioral Sciences University of Washington School of Medicine Seattle, WA United States; 2 Center for Child Health, Behavior and Development Seattle Children's Research Institute Seattle, WA United States; 3 Department of Pediatrics University of Washington School of Medicine Seattle, WA United States; 4 Department of Psychology University of California, Berkeley Berkeley, CA United States; 5 Department of Internal Medicine University of Washington School of Medicine Seattle, WA United States; 6 Clinical Research Division Fred Hutchinson Cancer Center Seattle, WA United States; 7 Institute of Psychiatry, Psychology, and Neuroscience Department of Psychology King’s College London London United Kingdom; 8 Department of Anesthesiology & Pain Medicine University of Washington School of Medicine Seattle, WA United States

**Keywords:** adolescent, young adult, cancer survivors, anxiety, attention bias, psychosocial impact of illness, randomized controlled trial, digital health, mobile apps

## Abstract

**Background:**

Adolescent and young adult cancer survivors face a high burden of psychological late effects, with cancer-related anxiety being a prevalent mental health concern. Despite the significant need for care, more than half of adolescent and young adult cancer survivors, who require psychosocial services, remain untreated. Digital health interventions offer a promising solution to bridge this care gap. Attention bias modification (ABM) is an evidence-based digital intervention for anxiety disorders. This intervention targets automatic and unconscious negative attention biases and retrains attention away from threat and toward neutral or positive stimuli. Recent research has successfully adapted ABM interventions for cancer survivors. However, ABM has not yet been adapted or tested for adolescent and young adult cancer survivors.

**Objective:**

This protocol describes a pilot randomized controlled trial designed to evaluate a novel digital anxiety intervention, the Attention Bias in Cancer survivors (ABCs) intervention, for adolescent and young adult cancer survivors.

**Methods:**

This is a single-site, 2-arm, pilot randomized controlled trial enrolling 60 cancer survivors aged 15 to 29 years. Participants will be randomized 1:1 to the ABCs intervention or a sham control condition. The ABCs intervention combines an ABM mobile intervention with daily gratitude and savoring SMS text messages (positive psychology prompts to savor positive emotions) over a 4-week period. The sham condition consists of sham ABM (showing the same cancer-related word stimuli as the active intervention condition but without attention retraining) and daily mood monitoring SMS text messages (prompts to report on current mood and stress levels). The primary objectives are to evaluate intervention feasibility (defined as ≥50% enrollment and ≥70% retention) and acceptability (defined by cutoff scores on the Client Satisfaction Questionnaire and System Usability Scale). Secondary exploratory outcomes include patient-reported measures of attention bias, anxiety, fear of recurrence, pain, resilience, and other psychosocial outcomes.

**Results:**

This study was funded in August 2023, and study recruitment began in November 2024. We have completed data collection as of February 2026. We anticipate that data analyses will be completed by September 2026. Manuscript preparation and submission are anticipated for December 2026.

**Conclusions:**

This pilot trial examines the feasibility and acceptability of a digital positive psychological intervention targeting anxiety in adolescent and young adult cancer survivors. Exploratory outcomes will inform sample size calculations for a future-powered multisite clinical trial. The ABCs intervention may provide scalable and accessible evidence-based psychosocial care and improve health outcomes.

**Trial Registration:**

ClinicalTrials.gov NCT06682039; https://clinicaltrials.gov/study/NCT06682039

**International Registered Report Identifier (IRRID):**

DERR1-10.2196/82665

## Introduction

### Background

Nearly 2 million adolescent and young adult cancer survivors reside in the United States [1]. In this study, adolescent and young adult cancer survivors are defined by their current developmental age (15-29 years) and include those diagnosed during childhood and adolescence and treated within pediatric cancer centers. This inclusive definition aligns with the Children’s Oncology Group framework for cancer survivorship care, which emphasizes that the psychosocial and medical needs of cancer survivors are based on their current developmental stage and navigation of transition-to-adulthood milestones [2]. Cancer profoundly disrupts normal developmental experiences and life transitions [3,4]. Consequently, adolescent and young adult cancer survivors are at elevated risk for long-lasting negative psychological late effects, with poorer health-related quality of life and psychosocial outcomes than their younger pediatric and adult counterparts [5]. Adolescent and young adult cancer survivors commonly struggle with reintegration into daily life after treatment; miss crucial social, educational, and vocational milestones; face disrupted peer and family relationships; navigate financial toxicity; and experience a forestalled transition to independence. All of these issues can be further exacerbated by discontinuation of comprehensive support from their oncology care team [6]. A seminal longitudinal population-based study (the AYA Health Outcomes and Patient Experience study) showed that more than half of adolescent and young adult cancer survivors, who require psychosocial services, remain untreated [7]. Barriers to psychosocial care include inadequate end-of-treatment transition care planning between pediatric and adult cancer centers and primary care settings, insufficient psychosocial screening and referrals to mental health providers, a shortage of psychosocial clinicians, and persistent mental health stigma [8,9].

Cancer-related anxiety is the most prevalent mental health problem, impacting one-third of adolescent and young adult cancer survivors [10-12]. Muzzin et al [13] aptly characterized this as a “living-dying” experience in which “a person never really ‘gets over’ cancer: it is a sword of Damocles that continues to hang over the individual and [their] family for the rest of the person’s life.” In addition to general anxiety, up to 85% of adolescent and young adults experience cancer-specific anxiety, most notably scan-associated anxiety and fear of cancer recurrence [11]. Cancer-related anxiety is associated with long-term negative outcomes such as poor quality of life, depression, distress, posttraumatic stress, substance use, sleep problems, fatigue, and pain [14]. Therefore, the identification and management of anxiety are important targets for follow-up care of adolescent and young adult cancer survivors.

Adopting a developmental science framework, “meeting teens where they are,” necessitates leveraging technology and a paradigm shift in traditional treatment norms [15]. Increasingly, mental health delivery models are being deployed in asynchronous mobile health formats. For adolescent and young adults, the internet serves as a primary platform for physical health and mental health–related information seeking and communication [16,17]. Adolescent and young adults prefer digital self-help interventions and may be reluctant to seek traditional forms of psychotherapy due to social stigma and discomfort discussing personal problems [16,18-20]. SMS text messaging interventions may be particularly appealing to younger generations who are accustomed to interacting on smartphones and using the internet [18,19,21,22].

Positive psychological interventions have demonstrated improvements in psychosocial outcomes for adolescent and young adult cancer survivors [23]. Such interventions, designed to increase positive emotions, have beneficial long-term impacts on emotion regulation, mental health, mindset broadening, coping resources, and functional outcomes [24]. Interventions that increase the frequency and duration of positive emotions through practices such as savoring (defined as the process of intentionally bringing focused attention to past, present, and future positive experiences, such as reminiscing about a happy memory or anticipating an upcoming vacation) and gratitude (defined as the process of appreciating the good in one’s life and ascribing their internal and external sources, such as writing a thank you note to someone who helped you) have been shown to decrease stress, promote psychological well-being, and improve health-related quality of life in the face of adversity (eg, a cancer diagnosis) [25-28]. Background literature suggests that positive psychological interventions involving gratitude and savoring activities increase treatment adherence [29,30] and have demonstrated efficacy and effectiveness of these interventions in systematic reviews [31-34]. Although few digital psychosocial interventions for adolescent and young adult cancer survivors currently exist, this is a burgeoning area of intervention research that is highly appealing and appropriate for adolescent and young adults [35-38]. To our knowledge, there are no existing adolescent and young adult cancer survivor–specific digital interventions that are designed to target anxiety, which is the most prevalent mental health symptom impacting adolescent and young adults.

Across pediatric and adult clinical populations alike, attention bias modification (ABM) is a widely used digital intervention for anxiety that has been adapted for a wide range of clinical populations and disease groups [39-41]. Systematic maladaptive patterns of information processing play a causal role in anxiety pathology [42,43]. Cognitive models of anxiety have consistently shown that clinically anxious individuals demonstrate an attention bias, meaning that they selectively attend to and have difficulty disengaging from negative information. Similarly, a systematic review has shown that cancer survivors demonstrate a negative attention bias [44]. For example, an individual with social anxiety may selectively attend to threatening stimuli in their environment, such as threatening facial expressions (eg, angry, judgmental, or disapproving), which can lead to sustained and increased anxiety and the avoidance of social situations [39,45-47].

ABM is a brief, self-guided digital intervention in which individuals complete repetitive association reaction-time tasks. These tasks target automatic and unconscious negative attention biases to retrain attention away from perceived threat and toward neutral or positive stimuli (ie, a modified dot-probe task) [48]. For example, ABM for social anxiety may involve a series of image pairs on a computer screen—one of a neutral or positive face and one of a threatening face. By rapidly and repeatedly training anxious individuals to disengage their attention from the threatening face and direct it toward the neutral or positive face, ABM implicitly modifies their negative attention bias.

ABM delivered digitally for the treatment of anxiety disorders (including social anxiety, generalized anxiety, and posttraumatic stress) has demonstrated moderate treatment effects [49-52]. The effectiveness of ABM is comparable to that of the gold-standard evidence-based treatment for anxiety (ie, cognitive behavioral therapy) [53]. A recent systematic review revealed that adult cancer survivors also experience negative attention biases with large effect sizes [44]. Recent research efforts have successfully adapted ABM for adult cancer survivors, demonstrating feasibility in pilot studies [54]. Notably, a pilot randomized controlled trial (RCT) of ABM in adult breast cancer survivors showed significant improvements in health-related anxiety and fear of cancer recurrence [55]. However, ABM has not yet been adapted or tested for adolescent and young adult cancer survivors.

This paper describes the protocol for an ongoing pilot RCT. This study aims to test the feasibility and acceptability of a novel digital positive psychological intervention for adolescent and young adult cancer survivors, the Attention Bias in Cancer survivors (ABCs) intervention, using a pilot RCT. The ABCs intervention consists of an ABM mobile intervention combined with a gratitude and savoring texting intervention. We hypothesize that the ABCs intervention will meet benchmarks for feasibility (enrollment and retention) and acceptability (user satisfaction and system usability).

### Study Aims

The primary aim of this study is to evaluate the feasibility and acceptability of a digital health intervention for adolescent and young adult cancer survivors. We hypothesize that the ABCs intervention will be feasible as measured by ≥50% enrollment of eligible patients and ≥70% retention of participants in both arms (with retention defined as completion of primary outcome measures after treatment). These benchmarks are based on our previous research and known challenges in the recruitment and retention of adolescent and young adult cancer survivors and background literature on expected enrollment and retention rates for remote digital health studies [56,57]. We hypothesize that the ABCs intervention will be acceptable, as measured by the Client Satisfaction Questionnaire (established cutoff score of ≥26 indicating satisfaction) [58-61] and the System Usability Scale (SUS; established cutoff score of ≥70 indicating usability) [62]. An exploratory aim is to examine preliminary efficacy assessed via validated patient-reported outcome measures after treatment.

## Methods

### Ethical Considerations

All study procedures were approved by the Seattle Children’s Hospital institutional review board (STUDY00004811). The trial is registered at ClinicalTrials.gov (NCT06682039). Informed consent (for individuals aged 18 to 29 years) or assent with parental consent (for individuals aged 15 to 17 years) will be obtained from all participants. Privacy and confidentiality protections are in accordance with institutional ethics guidelines. Participants will be compensated US $100 in electronic gift cards for completing a standardized survey assessment battery and attention bias assessment at T1 (baseline) and T2 (4 weeks after the intervention), each designed to be approximately 30 minutes long. Refer to Table 1 for the schedule of assessments. This protocol follows the SPIRIT (Standard Protocol Items: Recommendations for Interventional Trials) guidelines. The completed SPIRIT checklist is provided in Multimedia Appendix 1 [63].

**Table 1:** Schedule for outcome measures.

Measures	Baseline assessment (T1)	Posttreatment assessment (T2; 4 weeks)
Client Satisfaction Questionnaire-8		✓
System Usability Scale		✓
Treatment Experiences Questionnaire		✓
Attention bias assessment	✓	✓
Attention Bias Questionnaire	✓	✓
Hospital Anxiety and Depression Scale	✓	✓
Short Health Anxiety Inventory	✓	✓
Fear of Cancer Recurrence Inventory-Short Form	✓	✓
Connor-Davidson 10-item Resilience Scale	✓	✓
Kessler-6 Psychological Distress Scale	✓	✓
Pain frequency	✓	✓
PROMIS^a^ Pain Intensity	✓	✓
Child Activity Limitations Interview-9	✓	✓
Adolescent Sleep-Wake Scale-Short Form	✓	✓
Adolescent Insomnia Questionnaire	✓	✓
PROMIS NeuroQoL^b^ Cognitive Function-Short Form	✓	✓

^a^PROMIS: Patient-Reported Outcomes Measurement Information System.

^b^NeuroQoL: quality of life in neurological disorders.

### Trial Design

**Figure 1:**
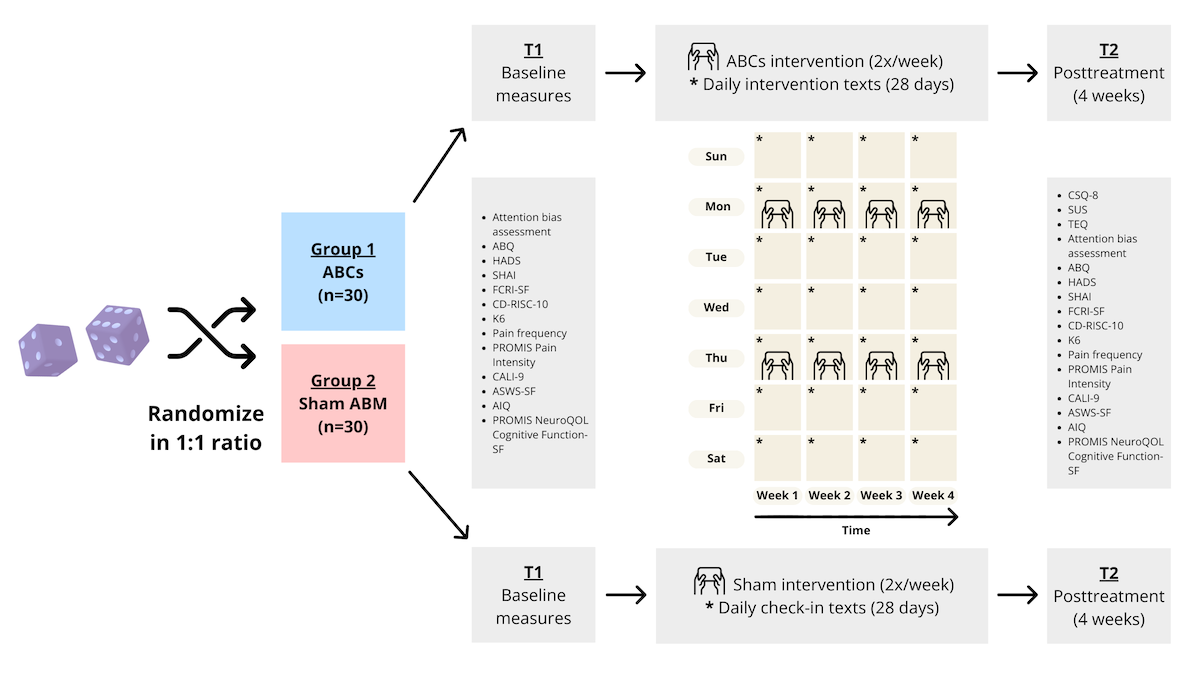
Timeline of study procedures for parallel, 2-arm, pilot randomized controlled trial. ABM: attention bias modification; ABQ: Attention Bias Questionnaire; AIQ: Adolescent Insomnia Questionnaire; ASWS-SF: Adolescent Sleep-Wake Scale–Short Form; CALI-9: child Activity Limitations Interview-9; CD-RISC-10: Connor-Davidson 10-item Resilience Scale; CSQ-8: Client Satisfaction Questionnaire-8; FCRI-SF: Fear of Cancer Recurrence Inventory-Short Form; HADS: Hospital Anxiety and Depression Scale; K6: Kessler-6 Psychological Distress Scale; NeuroQoL: quality of life in neurological disorders; PROMIS: Patient-Reported Outcomes Measurement Information System; SHAI: Short Health Anxiety Inventory; SF: Short Form; SUS: System Usability Scale; TEQ: Treatment Experiences Questionnaire.

This pilot RCT uses a parallel, 2-arm design (Figure 1). A total of 60 participants will be enrolled at a single site and then randomized in a 1:1 allocation ratio after completing baseline surveys. The randomization schedule will be stratified by age group (those aged 15 to 18 years and those aged 19 to 29 years). Age was selected as the stratification factor to account for developmental considerations and to ensure the appropriateness of the intervention design across developmental stages. Randomization was seeded based on the exact date and time of randomization for reproducibility. All randomization was conducted by the study coordinator and data manager in R (version 4.4; R Foundation for Statistical Computing) using the *blockrand* package [64]. Randomization was conducted using permuted varying block sizes of 2, 4, and 6. Participants will be assigned to either the active ABCs intervention condition (ABM and daily savoring and gratitude SMS text messages) or a sham control condition (sham ABM and daily mood SMS text messages). Participants will remain blinded to their study condition for the duration of the study. The principal investigator and the biostatistician responsible for data analysis are blinded to the study condition. The study coordinators responsible for study procedures could not be feasibly blinded to study conditions due to study management and logistics. However, the active treatment and sham treatment are entirely remotely and digitally delivered with no personnel involvement in the administration or facilitation of the intervention.

### Participants

Individuals are eligible for study participation if they are currently aged 15 to 29 years. Although the National Cancer Institute definition of adolescent and young adults includes ages 15 to 39 years, we targeted a narrower age range (15 to 29 years) to align with the pediatric and adolescent and young adult populations typically treated within pediatric cancer centers, such as Seattle Children’s Hospital, where this study is conducted. Eligibility criteria include a pediatric cancer diagnosis (inclusive of those diagnosed before the age of 15 years); completion of cancer treatment and in active follow-up cancer survivorship care at Seattle Children’s Hospital; proficiency in English (speaking, reading, and writing); having no cognitive limitations that would make participation difficult; and having access to a smartphone with SMS text messaging capabilities. adolescent and young adults are excluded from study participation if they are receiving active or curative treatment, are not proficient in English (speaking, reading, and writing), or are cognitively or physically unable to participate. Clinical research coordinators are identifying potential participants and conducting their prescreening assessments through clinic rosters and electronic medical records. Recruitment by clinical research coordinators occurs in person at clinic visits and remotely by phone, video, or text.

### Dissemination Plan

Study participants who have opted in will receive a layperson-accessible summary of study findings after we have completed primary data analysis. Deidentified study results will be disseminated to the scientific and clinical community through national and international conference presentations, peer-reviewed publications, and social media platforms.

### Intervention Conditions

#### ABCs Intervention (ABM and Daily Savoring and Gratitude SMS Text Messages)

The ABCs intervention integrates 2 distinct components: an ABM mobile intervention and daily gratitude and savoring SMS text messages.

#### Attention Bias Modification

We created the ABM intervention with word stimuli tailored to cancer-specific anxiety, worries, and fears (Multimedia Appendix 2). Intervention development consisted of an iterative human-centered co-design process to ensure that the stimuli were clinically relevant and developmentally appropriate for adolescent and young adult cancer survivors. Our team first generated a preliminary list of common cancer-specific negative stimuli. The stimuli word list was then formally reviewed by key stakeholders including 6 adolescent and young adult cancer survivors, 6 pediatric oncologists, 6 psychologists, 1 psychiatrist, 1 ethicist, and 2 social workers. Stakeholders evaluated the word stimuli for readability, emotional salience, and relevance to the adolescent and young adult cancer survivorship population. Stakeholders were also encouraged to propose new cancer-related threat stimuli. The stimuli word list was further refined based on stakeholder group consensus and finalized by the study team.

**Figure 2:**
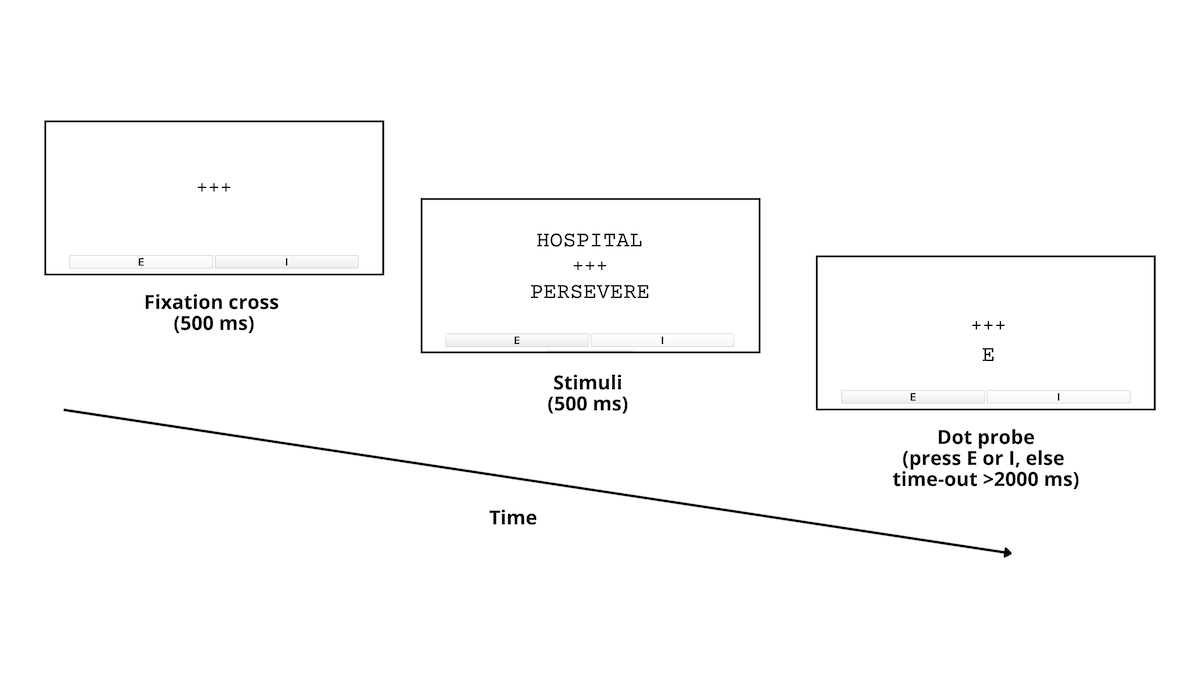
Example of a single dot-probe trial of the attention bias modification intervention.

Participants are instructed to complete ABM over a 4-week intervention period. ABM is completed using an app, Inquisit 6 Player (version 6.6.3; Millisecond Software LLC), to enable psychological tests remotely via mobile devices [65]. A description of active ABM intervention sessions is provided in Table 2. A single dot-probe trial is depicted in Figure 2. The total number of trials per ABM session was modeled after a large international multisite study of children and adolescents without cancer [66].

**Table 2:** Description of active attention bias modification (ABM) and sham ABM sessions.

	Active ABM	Sham ABM
Frequency of sessions	Twice per week	Twice per week
Duration of sessions	4 weeks	4 weeks
Single dot-probe trial (Figure 2)	A fixation cross appears for 500 ms. Paired negative and neutral (positive) stimuli appear for 500 ms. A dot-probe appears in a location *consistent with neutral (positive) stimuli*, disappearing once the participant presses the “left” (letter “E”) or “right” (letter “I”) key. If no key is pressed within 2000 ms, the program automatically advances to the next trial.	A fixation cross appears for 500 ms. Paired negative and neutral (positive) stimuli appear for 500 ms. A dot-probe is *equally likely to appear in a location consistent with either negative or neutral (positive) stimuli*, disappearing once the participant presses the “left” (letter “E”) or “right” (letter “I”) key. If no key is pressed within 2000 ms, the program automatically advances to the next trial.
Number of trials per session	120	120

#### Gratitude and Savoring SMS Text Messages

**Figure 3:**
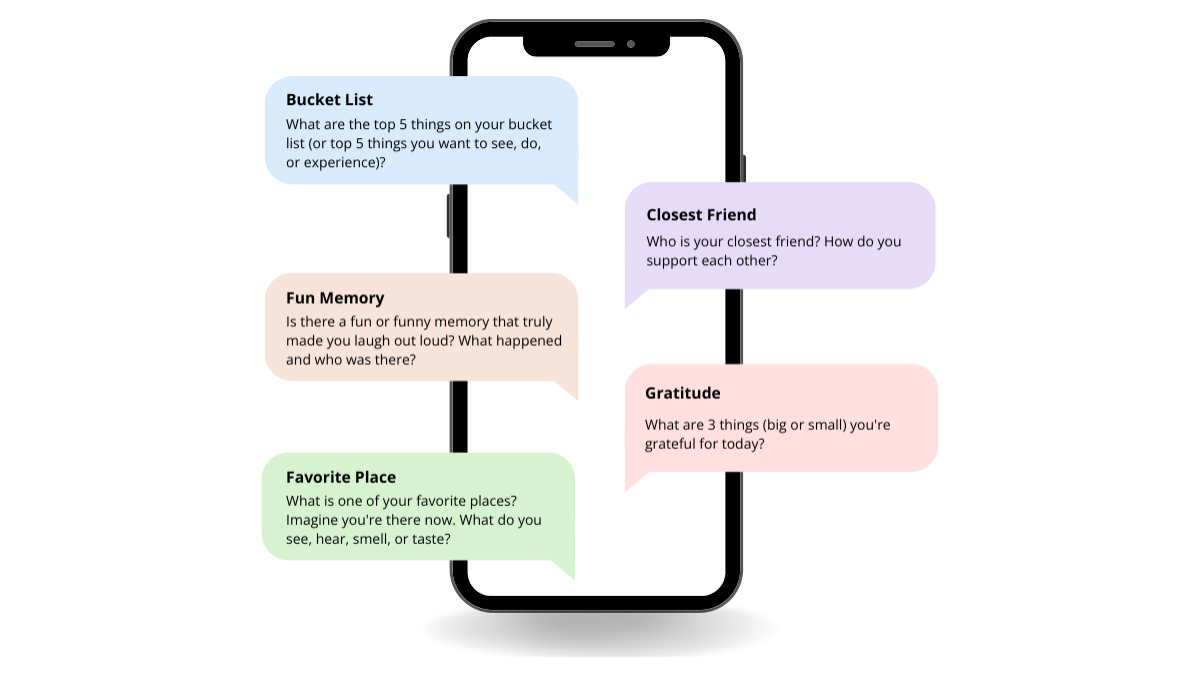
Example of daily savoring and gratitude intervention SMS text messaging prompts.

Automated interactive savoring and gratitude SMS text messages are sent to participants daily at a prespecified time of their preference, soliciting open-ended responses. Gratitude and savoring intervention SMS text messaging prompts encourage participants to engage in past, present, and future–oriented savoring of positive emotions related to people, places, and things for which they are grateful (Figure 3). Intervention prompts aim to extend and increase positive emotions by fostering a meta-awareness of positive experiences, including positive memory building, sensory-perceptual experiences, positive imagination, and reminiscing about past positive memories. Participants also respond to daily mood monitoring prompts: (1) How content, happy, or calm are you feeling right now? (2) How stressed are you feeling right now?

### Sham Control Condition (Sham ABM and Mood SMS Text Messages)

The sham control condition is designed to mirror the active intervention condition, providing a nontherapeutic sham ABM mobile intervention plus daily mood monitoring texts*.*

#### Sham ABM

Participants are instructed to complete sham ABM via the Inquisit app over a 4-week intervention period. Consistent with established ABM protocols, the sham condition uses the same negative threat stimuli as the active treatment condition *without* attention retraining toward neutral or positive stimuli, which is necessary to modify attention bias. This design ensures that the sham condition is identical to the active treatment condition in duration, task structure, and appearance. A description of sham ABM intervention sessions has been provided in Table 2.

#### Mood Monitoring SMS Text Messages

Automated interactive mood monitoring SMS text messages are sent to participants daily at a prespecified time of their preference. SMS text messages consist of the following prompts: (1) How content, happy, or calm are you feeling right now? (2) How stressed are you feeling right now?

### Primary Outcomes

#### Feasibility

Feasibility is defined by recruitment and retention rates. The intervention will be considered feasible if we achieve ≥50% enrollment of eligible patients and ≥70% retention across both study arms. We will report the eligibility rate, defined as the proportion of adolescent and young adults eligible to participate in the study out of all adolescent and young adults we attempted to contact. We will report the participation rate, defined as the proportion of eligible adolescent and young adults who enrolled in the study. Adherence to the 4-week ABCs intervention and the sham control condition will be monitored via the app backend and REDCap (Research Electronic Data Capture) SMS text messaging logs. For both the active treatment and sham conditions, we will track the number of ABM sessions completed and the number of text prompts answered. There are no established benchmarks for optimal digital intervention adherence, and such interventions are typically associated with low engagement. For the purpose of this pilot trial, we have defined intervention exposure as the completion of at least one ABM session and one SMS text message interaction. We will report on data completeness for all primary and exploratory outcome measures, aiming for ≥80% data completeness across patient-reported outcome measures at T1 (baseline) and T2 (after treatment).

#### Client Satisfaction Questionnaire-8

The Client Satisfaction Questionnaire-8 is an 8-item questionnaire used to assess the level of satisfaction with care. Items are scored on a Likert scale from 1 (low satisfaction) to 4 (high satisfaction) with different descriptors for each response point. Total scores range from 8 to 32, with higher scores indicating greater satisfaction (scores ≥26 indicate satisfaction). The Client Satisfaction Questionnaire-8 has been found to have high internal consistency and concurrent validity in inpatient and outpatient mental health settings [58-61].

#### System Usability Scale

The SUS is a well-validated and widely used 10-item scale to evaluate the perceived usability of digital interventions [62]. Items are rated on a 5-point Likert scale, reverse-scored as applicable, and converted to a 4-point scale, then multiplied by 2.5 to yield a total score ranging from 0 to 100; scores ≥70 demonstrate adequate usability.

### Exploratory Outcomes

#### Treatment Experiences Questionnaire

The Treatment Experiences Questionnaire is a measure developed by the senior author and has been used in digital health intervention trials conducted by our research team. This is a 2-item measure in which participants in both study arms will indicate whether they experienced any negative effects from the program and how severely the program affected them.

#### Attention Bias Assessment

Attention bias will be assessed using a dot-probe task administered via the Inquisit 6 Player app. Attention bias scores will be computed by subtracting the mean reaction time from trials in which probes replace negative stimuli from the mean reaction time of trials in which probes replace neutral or positive stimuli. A positive score will indicate an attentional bias toward threatening stimuli, and negative scores will indicate an attentional bias toward nonthreatening stimuli.

#### Attention Bias Questionnaire

The Attention Bias Questionnaire consists of 9 items, reflecting the subjective experience of attention bias toward threats, with two subscales: engagement with threat and difficulty disengaging from threat. High scores in the Attention Bias Questionnaire are correlated with trait anxiety, social anxiety, posttraumatic stress disorder, and depression. It has been validated in individuals aged 18 years and older, with high internal consistency (Cronbach α=0.90) [67].

#### Hospital Anxiety and Depression Scale

The Hospital Anxiety and Depression Scale is a 14-item measure that assesses symptoms of anxiety and depression, respectively, in patients with serious illness. It has been validated in adolescent and young adults with chronic illness and cancer survivors, with excellent reliability (Cronbach α=0.83) [68-71]. Items are scored from 0 to 3 (subscale range 0-21), with scores ≥8 categorized as borderline abnormal and ≥11 categorized as abnormal.

#### Short Health Anxiety Inventory

The Short Health Anxiety Inventory contains 14 items that assess health anxiety independent of physical health status. Items assess worry about one’s health, awareness of bodily sensations and changes, and the feared consequences of having an illness. It can be used in both healthy and physically ill individuals, including those who were temporarily sick or diagnosed with a serious and chronic illness. The scale has been validated in clinical and nonclinical samples [72,73].

#### Fear of Cancer Recurrence Inventory-Short Form

The Fear of Cancer Recurrence Inventory-Short Form is a widely used and validated 9-item measure that evaluates the presence and severity of intrusive thoughts associated with fear of cancer recurrence [74-76]. Each item is rated on a Likert scale ranging from 0 (“not at all” or “never”) to 4 (“a great deal’ or “all the time”). A higher score indicates higher levels of fear of cancer recurrence.

#### Connor-Davidson 10-Item Resilience Scale

The Connor-Davidson 10-item Resilience Scale is a validated and widely used instrument to measure inherent resilience [77]. Questions revolve around personal problem-solving and approaches to adversity. The 10-item instrument has high internal consistency (Cronbach α=0.85) and has been used in diverse populations including adolescents, parents, and patients with cancer [78]. Each item consists of a 5-point Likert scale (scored from 0 to 4), for a total of 40 points, with higher scores reflecting greater resilience.

#### Kessler-6 Psychological Distress Scale

This 6-item scale measures the level of psychological distress experienced in the past month. The instrument strongly discriminates between community cases and noncases of the *Diagnostic and Statistical Manual of Mental Disorders, Fifth Edition* (*DSM-5*) psychiatric disorders such as serious emotional distress or serious mental illness. It has been extensively cross-validated, including among adolescents [79,80]. Responses are scored on a 5-point Likert scale, generating a range of 0 to 24 points. Previous studies have shown that scores ≥7 are consistent with “high” distress, and those ≥13 meet criteria for serious or debilitating psychological distress.

#### Pain Frequency

Three questions will assess pain frequency (6-item or 7-item Likert scale ranging from “not at all” to “daily”), pain intensity (11-point Numeric Rating Scale, 0-10), and pain interference (11-point Numeric Rating Scale, 0-10).

#### Patient-Reported Outcomes Measurement Information System Pain Intensity

The Patient-Reported Outcomes Measurement Information System (PROMIS) are publicly available, rigorously tested, and well-validated instrument supported by the National Institutes of Health Common Fund. The PROMIS Pain Intensity instrument assesses how much a person hurts [81].

#### Child Activity Limitations Interview-9

The Child Activity Limitations Interview-9 is a brief measure for assessing activity limitations in children and adolescents with chronic pain. This measure has demonstrated good internal consistency and high cross-informant reliability [82].

#### Adolescent Sleep-Wake Scale–Short Form

The Adolescent Sleep-Wake Scale–Short Form is a 10-item measure of behavioral sleep patterns that has been validated in general adolescent and young adult populations [83,84]. The measure provides an overall sleep quality score and 3 subscale scores: going to bed, falling asleep and reinitiating sleep, and returning to wakefulness. Items are rated on a 6-point Likert scale and averaged to compute subscale and total measure scores. Total scores range from 1 to 6, with higher scores indicating better sleep quality.

#### Adolescent Insomnia Questionnaire

The Adolescent Insomnia Questionnaire is a 13-item screening measure of insomnia in adolescents. The measure has demonstrated strong reliability and high internal consistency in study samples up to the age of 19 years [85]. Items are rated on a 5-point Likert scale. Total scores range from 0 to 52, with higher scores indicating more insomnia symptoms.

#### PROMIS Quality of Life in Neurological Disorders Cognitive Function-Short Form

The PROMIS NeuroQoL (quality of life in neurological disorders) Cognitive Function-Short Form measures perceived difficulties in everyday cognitive abilities such as memory, attention, and decision-making [86].

### Data Analysis

The primary aim of this study is to determine the feasibility and acceptability of the ABCs intervention. Feasibility will be defined by meeting the following criteria: (1) ≥50% enrollment of eligible patients and (2) ≥70% retention of participants in both arms. Acceptability will be defined as (1) an average score ≥26 on the Client Satisfaction Questionnaire and (2) an average score ≥70 on the SUS.

All participants who complete baseline surveys and are randomized will be included in intent-to-treat analyses to explore the preliminary efficacy of all patient-reported outcome measures in our assessment battery. Preliminary efficacy will be examined using 2-sample comparisons and regression models. Two-sample comparisons will be based on 2-tailed *t* tests or chi-square tests, depending on the type of outcome. The regression models aim to assess the intervention’s effect on continuous patient-reported outcomes while controlling for baseline scores and other potential confounders. Potential covariates include age, gender, cancer diagnosis, and other sociodemographic or disease-related or treatment-related characteristics previously shown to influence psychosocial outcomes. All statistical analyses will be performed in the R software [64].

## Results

This project was funded in August 2023. The study launch date (date of first participant enrolled) was November 8, 2024. Data collection has been completed as of February 2026. After data collection, analyses are expected to be completed by September 2026. Dissemination of findings and manuscript submission are planned for December 2026.

## Discussion

### Anticipated Findings

This manuscript describes the protocol for a pilot RCT designed to evaluate the feasibility and acceptability of the ABCs intervention, which is a novel digital intervention tailored for adolescent and young adult cancer survivors. The ABCs intervention is specifically designed to target anxiety, addressing a critical gap in cancer survivorship care. We hypothesize that the ABCs digital intervention will demonstrate feasibility and acceptability within the adolescent and young adult cancer survivor population. The exploratory patient-reported outcomes data collected from this pilot trial will provide effect size estimates to inform power calculations and sample sizes for future multisite efficacy trials.

### Comparison to Previous Work

Digital health interventions offer a unique opportunity to address existing access care gaps by using technologies that align with adolescent and young adult communication and learning preferences. The ABCs intervention builds on a robust body of literature establishing ABM as a digital anxiety intervention across the age continuum and in various disease groups [39-41]. A previous systematic review has confirmed that cancer survivors, such as individuals with clinical anxiety disorders, demonstrate a negative attention bias [44]. However, existing digital psychosocial interventions for adolescent and young adult cancer survivors have not been designed to target anxiety symptoms [35-38]. To our knowledge, this protocol represents the first study to design and test a digital anxiety intervention for adolescent and young adult cancer survivors, targeting cognitive mechanisms of anxiety [42,43].

### Strengths

A primary strength of this study is the use of a widely used digital anxiety intervention, ABM, that we have adapted for adolescent and young adult cancer survivors. The development of the ABCs intervention followed a rigorous user-centered co-design approach involving a multidisciplinary group of stakeholders. The digital deployment model allows for remote delivery and scalability and helps overcome the access care gap that adolescent and young adult cancer survivors face when seeking traditional mental health services.

### Limitations

Our study has several anticipated limitations. First, this is a single-site pilot trial, which limits the generalizability of findings. However, a strength of this approach is the presence of preexisting relationships with the clinic to ensure project completion and the collection of pilot data for future clinical trials. Second, cognitive late effects may present difficulties in engaging with ABM, which consists of repetitive reaction-time association tasks. Although not yet tested in adolescent and young adult cancer survivors, findings from attention bias assessment and ABM studies in pediatric medical populations and adult breast cancer survivors support its potential feasibility. On the basis of the results of this ongoing trial and participant feedback, we may vary stimulus presentation times for future intervention refinement.

### Future Directions

Findings from this pilot trial will inform a larger multisite hybrid effectiveness-implementation trial. This is a critical next step in digitizing evidence-based positive psychological interventions and co-designing for engagement and sustainability. This study and future studies building on this work aim to expand the adoption and reach of evidence-based mobile health psychosocial interventions.

## Appendix

Multimedia Appendix 1 SPIRIT (Standard Protocol Items: Recommendations for Interventional Trials) 2025 checklist.

Multimedia Appendix 2 Attention bias modification word stimuli list for the Attention Bias in Cancer survivors intervention.

## Abbreviations

ABM: attention bias modification
ABCs: Attention Bias in Cancer survivor
DSM-5: Diagnostic and Statistical Manual of Mental Disorders-5
NeuroQoL: quality of life in neurological disorders
PROMIS: Patient-Reported Outcomes Measurement Information System
RCT: randomized controlled trial
REDCap: Research Electronic Data Capture
SPIRIT: Standard Protocol Items: Recommendations for Interventional Trials
SUS: System Usability Scale

## Data Availability

Deidentified human subjects data from this research study will be banked indefinitely. Data will be made available upon reasonable request to other researchers who seek approval from the research team via email to the principal investigator (NL).
