# Supplementary material for: Digital Attention Bias in Cancer Survivors Intervention for Adolescent and Young Adult Cancer Survivors: Protocol for a Pilot Randomized Controlled Trial
Source: JMIR Res Protoc. 2026 Feb 25;15:e82665. doi: 10.2196/82665 (PMC12980062; doi:10.2196/82665)
Supplement: Multimedia Appendix 2 [file resprot_v15i1e82665_app2.docx]

**Supplemental Appendix 2.** ABM word stimuli list for ABCs intervention.

| CANCER | LUMP | TUMOR | CHEMOTHERAPY |
| --- | --- | --- | --- |
| RADIATION | CANCEROUS | BIOPSY | INCURABLE |
| INFECTION | NAUSEA | AMBULANCE | CLINIC |
| DIAGNOSIS | DISEASE | DOCTOR | CONTRACTED |
| EXHAUSTED | EXPOSURE | HURT | ANTIBODY |
| BLOOD | BACTERIA | CHRONIC | DRUG |
| SPECIMEN | DOSE | DEATH | EMERGENCY |
| FATAL | HOSPITAL | ILLNESS | INJECTION |
| LAB | COLLAPSE | INJURY | PARALYSIS |
| ACHE | PAIN | SICK | ABNORMAL |
| SURGERY | TRANSPLANT | INVASIVE | MASS |
| RECURRENCE | RELAPSE | IMMUNOTHERAPY | HOSPICE |
| RISK | STAGE | NEEDLE | MEDICAL |
| SCAR | WEAK | TIRED | SWELLING |
| UNRESPONSIVE | FUNERAL | AMPUTATE | ONCOLOGY |
| LOSS | SCANS | ICU | ADMISSION |
| IMAGING | PLATELETS | INFERTILITY | COMPLICATIONS |
| MEDICATION | GERMS | AGGRESSIVE | PALLIATIVE |
| METASTATIC | TEST | PROCEDURE | INFUSION |
| TRIAL | SEPSIS | FAINT | FALL |
| UNCONSCIOUS | COMA | RARE | INTUBATE |
| TRANSFUSION | PORT | ALLERGIC | MRI |
| ATTACK | DYING | GRIEF | COFFIN |
| UGLY | HOPELESS | ISOLATION | ALONE |
| SAD | FAIL | TENSE | WORRIED |
| DISORDER | TRAGEDY | DISASTER | UNHAPPY |
| UNHEALTHY | TROUBLE | STRESSFUL | HEADACHE |
| DIE | TRAUMA | REJECTION | UNCERTAINTY |
| UNEASE | BLAME | SUFFER | ANGRY |
| VIRUS | GENETIC | LOSER | WHEELCHAIR |
